# High-dose inhaled nitric oxide for severe pneumonia caused by multidrug-resistant bacteria: a randomized controlled trial protocol

**DOI:** 10.1080/07853890.2026.2719854

**Published:** 2026-08-18

**Authors:** Penglei Yang, Jie He, Jun Yuan, Lina Yu, Yongjuan Wang, Qihong Chen

**Affiliations:** ^a^ Department of Critical Care Medicine, Jiangdu People’s Hospital Affiliated to Yangzhou University, Yangzhou, China; ^b^ Department of Emergency Medicine, Yangzhou Jiangdu Traditional Chinese Medicine Hospital, Yangzhou, China

**Keywords:** Multidrug-resistant bacteria, severe pneumonia, inhaled nitric oxide, randomized controlled trial, study protocol

## Abstract

**Introduction:**

Severe pneumonia caused by multidrug-resistant (MDR) bacteria is associated with high mortality. Inhaled nitric oxide (NO) has antimicrobial activity, but clinical evidence in MDR severe pneumonia remains limited. This trial will evaluate the efficacy and safety of adjunctive high-dose inhaled NO in patients with severe pneumonia associated with MDR bacteria.

**Patients and methods:**

This prospective, single-center, double-blind randomized controlled trial will be conducted at Jiangdu People’s Hospital Affiliated with Yangzhou University from January 2026 to June 2027. Patients with suspected or confirmed severe pneumonia caused by MDR bacteria will be randomized 1:1 to standard treatment plus inhaled NO (200 ppm, twice daily for 30 min/session for 5 days) or standard treatment plus sham inhalation (0 ppm NO). The primary outcome is the change in Clinical Pulmonary Infection Score (CPIS) from baseline to day 7. Secondary outcomes include mortality, 28-day ventilator-free days, vasopressor-free days, antibiotic-free days, ICU-free days, and hospital-free days, CPIS and Sequential Organ Failure Assessment scores, microbiological eradication, citrullinated histone H3 and cell-free DNA concentrations, and ratio of arterial partial pressure of oxygen to fraction of inspired oxygen (PaO_2_/FiO_2_) ratios. Safety outcomes include methemoglobin and NO_2_ thresholds, serum creatinine, airway hyperreactivity, and acute kidney injury. Primary and secondary outcomes will be analyzed in confirmed MDR participants; safety will be assessed in participants receiving at least one study-gas administration.

**Discussion:**

This protocol tests a mechanistically plausible adjunctive therapy for MDR severe pneumonia. It will explore whether high-dose NO affects neutrophil extracellular trap activity.

## Introduction

Multidrug-resistant (MDR) bacterial infection is one of the most important threats to global public health and remains a major unresolved problem in critical care medicine. By 2050, infections caused by MDR bacteria are projected to cause approximately 10 million deaths worldwide each year [1]. Pneumonia is among the most common types of infection. MDR bacterial infection substantially increases the risk of death in patients with pneumonia, particularly among those requiring mechanical ventilation [2].

Nitric oxide (NO) is a colorless, non-irritating gas that is lipid soluble, widely distributed in human tissues, and able to diffuse rapidly across cell membranes. As an intercellular signaling molecule, NO participates in the regulation of multiple physiological processes. When delivered locally at high-dose, inhaled NO also has antimicrobial activity and may provide a potential therapeutic approach for MDR infection [3]. In animal models, short-term high-dose inhaled NO safely and effectively reduced the pulmonary burden of MDR bacteria in mice [4]. Multiple clinical studies have reported that high-dose Nitric Oxide inhalation (160–240 ppm) may exert antimicrobial activity and is associated with reductions in pulmonary bacterial burden [5–7]. A randomized controlled trial reported that inhaled NO at 200 ppm reduced the incidence of hospital-acquired pneumonia after cardiac surgery from 24.3% to 5.4% [8]. Other studies have shown that inhaled NO can improve oxygenation indices in patients with coronavirus disease 2019 [9–11]. A recent clinical study also reported that high-dose inhaled NO did not increase adverse reactions or complications, supporting its potential clinical safety [12] Recently, Yu et al. [13] reported that inhaled NO at 300 ppm exhibited antibacterial activity against MDR bacteria. In this study, no serious adverse events were observed among 10 healthy adults and two critically ill patients [13]. Although high-dose inhaled NO may exhibit promising antibacterial activity against MDR bacteria, clinical evidence regarding its use in critically ill patients with MDR pneumonia remains lacking.

Patel et al. [14] and Manda-Handzlik et al. [15] reported that NO promotes the generation of reactive species and stimulates the release of neutrophil extracellular traps (NETs), while peroxynitrite may facilitate NET formation. Experimental studies have further suggested that high-dose inhaled NO may enhance neutrophil chemotaxis and activation, neutrophil extracellular trap release, and bacterial killing [14–16]. However, these effects have not yet been demonstrated in humans. Because MDR severe pneumonia has high mortality and lacks effective adjunctive treatments, high-dose inhaled NO is a plausible therapeutic strategy. However, clinical data in patients with severe pneumonia associated with MDR bacteria remains insufficient.

This prospective randomized controlled trial will evaluate adjunctive high-dose inhaled NO therapy in mechanically ventilated patients with severe pneumonia caused by MDR bacteria. The trial will evaluate the efficacy and safety of high-dose inhaled NO therapy by assessing the Clinical Pulmonary Infection Score (CPIS), mortality, bacterial eradication rates, methemoglobin (MetHb) levels, and markers of kidney injury.

## Patients and methods

### Study design

**Figure 1.**
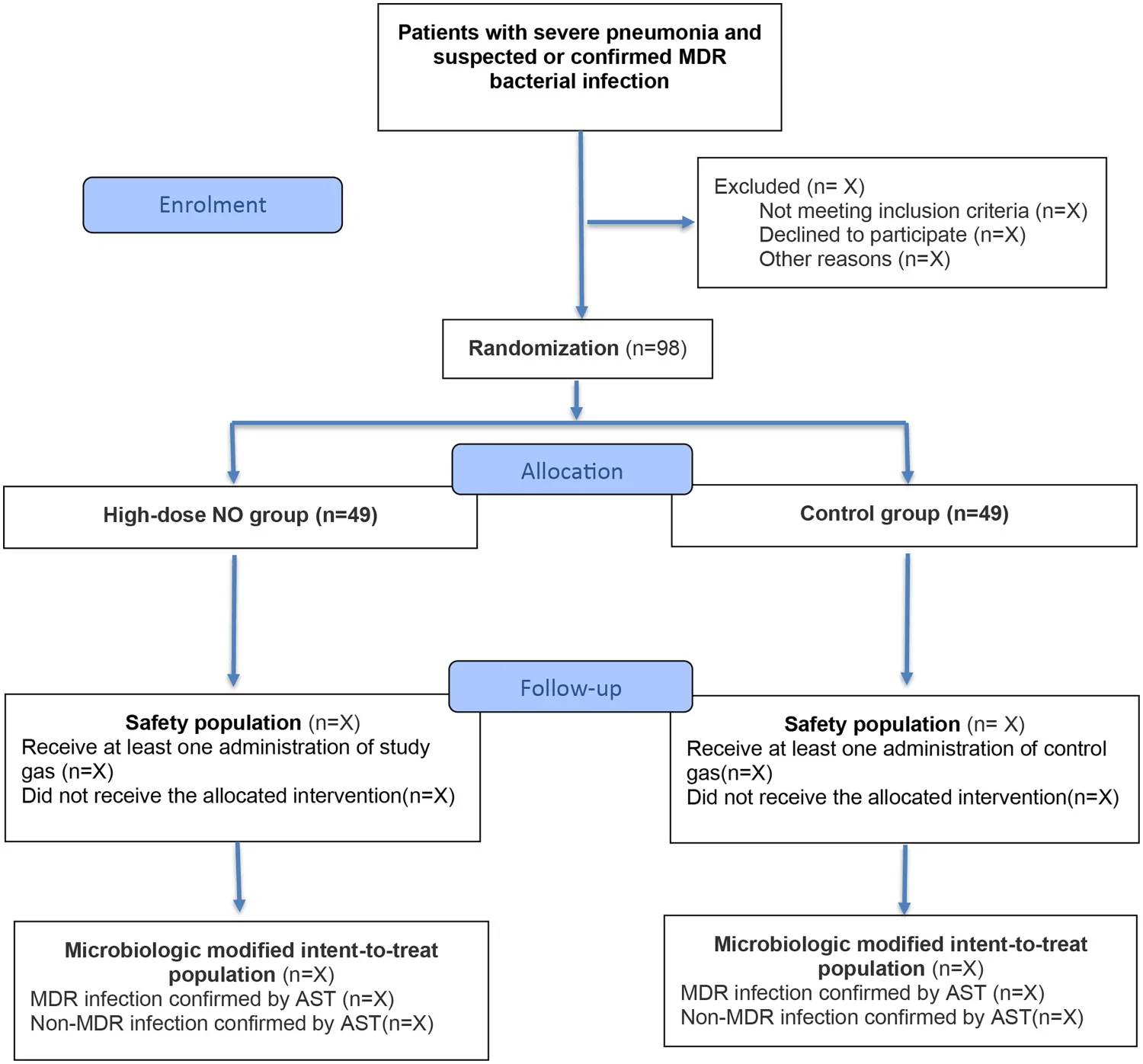
flow diagram of this trial. AST: Antimicrobial susceptibility testing; MDR: Multidrug-resistant; NO: Nitric oxide.

This prospective, single-center, double-blind, randomized controlled trial will enroll consecutive eligible patients admitted to Jiangdu People’s Hospital Affiliated with Yangzhou University between January 2026 and June 2027 with suspected or confirmed severe pneumonia caused by MDR bacteria. The study flow is shown in Figure 1. The protocol follows the SPIRIT statement (Table 1; Table S1) [17].

**Table 1.** Schedule of patient enrollment, study interventions, and outcome assessment according to SPIRIT statement.

			Study period									
	Enrollment		Post-randomization									
Timepoint	Admitted to the ICU	Suspected or confirmed MDR bacteria	Day1	Day2	Day3	Day4	Day5	Day6	Day7	ICU discharge	Hospital discharge	Day28
**Enrollment**												
Eligibility screen	X											
Inclusion criteria	X											
Written informed consent		X										
Exclusion criteria		X										
Baseline data		X										
Randomization		X										
**Study interventions**												
High-dose Nitric Oxide Inhalation		X	X	X	X	X	X	X	X			X
Control		X	X	X	X	X	X	X	X			
**Assessment**												
Change in CPIS			X		X				X			
ICU mortality rate										X		
Hospital mortality											X	
28-Day mortality rate												X
Ventilator-free days at day 28												X
Antibiotic-free days at day 28												X
SOFA score			X		X				X			
Citrullinated histone H3			X						X			
Cell-free DNA			X						X			
PaO_2_/FiO_2_			X	X	X	X	X	X	X			
Creatinine			X	X	X	X	X	X	X			
Methemoglobin			X	X	X	X	X	X	X			
NO₂			X	X	X	X	X	X	X			
AHR events			X	X	X	X	X	X	X			
AKI stages			X	X	X	X	X	X	X	X	X	

AHR events: airway hyperresponsiveness events; AKI: acute kidney injury; ICU: intensive care unit; MDR: multidrug-resistant; NO_2_: nitrogen dioxide; DNA: deoxyribonucleic acid; PaO_2_/FiO_2_: ratio of arterial oxygen partial pressure to fractional inspired oxygen; SOFA: Sequential Organ Failure Assessment.

### Ethics and registration

The study protocol was approved by the Ethics Committee of Jiangdu People’s Hospital Affiliated to Yangzhou University on 15 August 2025 (approval number: YJRY-2025-K-022). The trial was registered with the Chinese Clinical Trial Registry on 11 November 2025 (registration number: ChiCTR2500112185). All procedures will be conducted in accordance with the Declaration of Helsinki. Written informed consent will be obtained from each participant or legally authorized representative before enrollment.

### Participants

Eligible patients must meet all the following criteria:

1. Age 18 years or older.
2. Diagnosis of severe pneumonia within 24 h before enrolment.
3. Requirement for invasive mechanical ventilation.
4. Suspected or confirmed infection caused by multidrug-resistant (MDR) bacteria; confirmation may be based on sputum or bronchoalveolar lavage fluid (BALF) culture.
5. Written informed consent provided by the patient or a legally authorized representative.

Informed by previous studies by Di Fenza et al. [18] and Kalashnikova et al. [8], patients will be excluded if they meet any of the following criteria:

1. Current participation in another clinical trial;
2. Active pulmonary malignancy or a history of lung transplantation or pulmonary resection;
3. Severe burns involving >40% of the total body surface area;
4. Severe hepatic or renal dysfunction, defined as Child–Pugh class C hepatic impairment or an estimated glomerular filtration rate <30 mL/min;
5. Current receipt of renal replacement therapy;
6. Previous inhaled NO therapy before enrolment;
7. Patients with methemoglobinemia;
8. Major bleeding at screening.

#### Definition of pneumonia

Pneumonia is defined as the presence of a new or progressive pulmonary infiltrate on chest imaging, together with at least one of the following clinical features of infection: (1) new-onset cough or sputum production, or worsening of pre-existing respiratory symptoms, with or without purulent sputum, pleuritic chest pain, dyspnea, or hemoptysis; (2) fever; or (3) a peripheral white blood cell count >10 × 10^9^/L or <4 × 10^9^/L, with or without a left shift.

#### Definition of severe pneumonia

Severe pneumonia is defined according to the ATS/IDSA criteria. Major criteria are respiratory failure requiring invasive mechanical ventilation and septic shock requiring vasopressor support. Minor criteria are a respiratory rate ≥30 breaths/min, a PaO_2_/FiO_2_ ratio ≤250 mmHg, multilobar infiltrates, confusion or disorientation, blood urea nitrogen ≥20 mg/dL, leukopenia (white blood cell count <4,000 cells/μL), thrombocytopenia (platelet count <100,000/μL), hypothermia (core body temperature <36 °C), and hypotension requiring aggressive fluid resuscitation. Severe pneumonia is diagnosed in the presence of at least one major criterion or at least three minor criteria [19].

#### Definition of MDR bacteria

MDR bacteria are defined as bacteria resistant to three or more commonly used classes of antimicrobial agents [20]. Common clinically important MDR pathogens include carbapenem-resistant *Acinetobacter baumannii* (CRAB), carbapenem-resistant *Pseudomonas aeruginosa* (CRPA), difficult-to-treat-resistant *P. aeruginosa* (DTR-PA), carbapenem-resistant Enterobacterales (CRE), *Stenotrophomonas maltophilia*, and methicillin-resistant *Staphylococcus aureus* (MRSA), and other MDR pathogens.

**Suspected MDR infection** is defined as the presence of at least one of the following: (1) previous respiratory isolation of methicillin-resistant *Staphylococcus aureus* (MRSA) or *Pseudomonas aeruginosa*; (2) hospitalization and exposure to intravenous antibiotics within the preceding 90 days; or (3) rapid microbiological testing suggestive of MDR pathogens [19,21,22].

#### Definition of major bleeding

1. Fatal bleeding, 2. Symptomatic bleeding in a critical area or organ, such as intracranial, intraspinal, intraocular, retroperitoneal, intra-articular or pericardial, or intramuscular with compart-ment syndrome,3. Bleeding causing a fall in hemoglobin level of 20 g/L) or more, or leading to transfusion of two or more units of whole blood or red cells [23].

#### Randomization and blinding

Participants will be randomly assigned in a 1:1 ratio to the high-dose inhaled NO group or the control group using a computer-generated simple randomization sequence. Allocation concealment will be maintained by an independent unblinded research staff member who is not involved in participant screening, clinical care, outcome assessment, data collection, or statistical analysis and who will retain the randomization codes.

Both groups will use identical NO delivery devices, respiratory circuit connections, treatment frequencies, and treatment durations. In the high-dose inhaled NO group, the device will operate in its standard mode to deliver the protocol-specified NO concentration. In the control group, the device will operate in a sham NO research mode. This mode will maintain the same user interface, operating procedures, and display of study-related parameters as in the intervention group, but will deliver gas containing no NO (NO concentration, 0 ppm).

For each treatment session, the independent unblinded research staff member will perform device connection, mode selection, parameter setting, and operational verification. This staff member will not participate in clinical care, outcome assessment, data collection, or statistical analysis and will not disclose device mode or treatment allocation to other study personnel or the clinical team. Participants, treating physicians, bedside clinical staff, outcome assessors, data collectors, and statisticians will remain blinded to treatment allocation.

#### Emergency unblinding criteria

Emergency unblinding will be permitted only for a life-threatening medical event or an event that may result in irreversible harm, when the investigator determines that knowledge of the study gas received by the participant is necessary for immediate resuscitation or subsequent critical treatment decisions.

Individual emergency unblinding may be considered if any of the following occurs:

1. Uncontrolled respiratory or hemodynamic deterioration, including persistent hypoxemia, urgent escalation of respiratory support, circulatory shock, severe arrhythmia, or cardiac arrest, when knowledge of whether the participant received inhaled NO would alter immediate clinical management.
2. A suspected serious adverse reaction or serious unexpected medical event related to study gas, for which treatment allocation remains necessary to guide further management after discontinuation of the study gas and standard clinical treatment.
3. Any other emergency that is life-threatening or may result in irreversible harm, when the investigator determines that failure to unblind would compromise participant safety.

#### Interventions

Participants in the intervention group will receive iNO at 200 ppm two times daily, with each session lasting 30 min, for 5 days, beginning within 24 h of enrollment [8]. Both the intervention and control groups will receive standard treatment according to relevant guidelines for severe pneumonia. Standard treatment may include empirical and targeted anti-infective therapy, fluid resuscitation, vasoactive drugs, lung-protective ventilation, organ support, nutritional support, and immunomodulatory therapy as clinically indicated. Once antimicrobial susceptibility results are available, participants in both groups with confirmed MDR bacterial infection will continue the protocol-directed study intervention. For participants in whom MDR bacterial infection is not confirmed, the study intervention administered solely for research purposes will be discontinued in both groups.

Inhaled NO will be administered using the NOwill N300 Nitric Oxide Generation and Delivery System (Noling Biotechnology Co., Ltd., Nanjing, China). All device installation, mode selection, circuit connection, pre-treatment safety checks, and monitoring during treatment will be performed exclusively by designated independent research personnel who have completed manufacturer training and are qualified to operate the system. These personnel will not participate in participant screening, routine clinical care, outcome assessment, data collection, or statistical analysis.

At enrolment, inhaled NO will be delivered through the mechanical ventilator circuit. If a participant is extubated during the treatment period, inhaled NO will be administered through an interface compatible with the selected sequential oxygen therapy modality.

The NOwill N300 system may be connected to invasive ventilators, noninvasive ventilators, high-flow oxygen therapy systems, nasal cannulae, or face masks. For participants receiving invasive mechanical ventilation, the NO delivery device will be integrated into the ventilator circuit at the gas outlet, with a flow sensor and NO injection port installed. The NO injection port will be positioned upstream of the humidification chamber to enable NO delivery during the inspiratory phase.

The NO sampling port will be placed on the inspiratory limb close to the patient and at least 15 cm from the Y-piece. Connections to noninvasive ventilation and high-flow oxygen therapy systems will follow the same principles. During noninvasive ventilation, the NO sampling port will be positioned at least 15 cm from the exhalation valve.

Before and throughout each treatment session, dedicated study personnel will continuously monitor inspired NO concentration, NO_2_ concentration, oxygen concentration, device operating status, and alarm notifications.

#### Temporary suspension of a treatment session

The study-gas treatment session will be immediately suspended if any of the following occurs:

1. Methemoglobin (MetHb) will be measured 15 min after initiation of each treatment session and at the end of treatment. If MetHb exceeds 7%, the current inhaled NO treatment will be stopped.
2. The inhaled NO_2_ concentration exceeds the prespecified threshold of 3 ppm.
3. A device, respiratory circuit, gas-delivery, or monitoring-system malfunction occurs and cannot be immediately corrected.
4. Clinically significant active bleeding, marked hemodynamic instability, bronchospasm, or unexplained deterioration in oxygenation potentially related to the study gas occurs, as judged by the treating physician.

#### Permanent discontinuation of study-gas treatment

Study-gas treatment administered for research purposes will be permanently discontinued if any of the following occurs:

1. Completion of the planned 5-day treatment course.
2. Antimicrobial susceptibility results do not confirm MDR bacterial infection.
3. Withdrawal of informed consent.
4. MetHb ≥7% or inhaled NO_2_ ≥3 ppm on two occasions.
5. A suspected serious adverse reaction related to the study gas.
6. The treating physician or principal investigator judges that continued intervention poses an unacceptable risk.

Participants who discontinue the intervention will continue scheduled follow-up and will be included in the intention-to-treat analysis.

#### Trial sponsor, study intervention supply

The trial sponsor is Jiangdu People’s Hospital Affiliated to Yangzhou University. The sponsor is responsible for overall trial oversight, regulatory and ethics submissions, maintenance of essential trial documentation, and ensuring that the trial is conducted in accordance with the approved protocol and applicable regulations.

This investigator-initiated trial is funded by the Yangzhou City Basic Research Program (Joint Special Project), Health and Wellness Category (2025-3-45). The funder has no role in the design of the study; collection, management, analysis, or interpretation of data; decision to submit the results for publication; or preparation of the manuscript.

Both the intervention and control groups will receive study-gas treatment using the NOwill N300 Nitric Oxide Generation and Delivery System (Noling Biotechnology Co., Ltd., Nanjing, China). The equipment will be provided by the hospital, and the manufacturer will provide software support only. The manufacturer will not participate in protocol development, participant recruitment, clinical care, randomization, data collection, outcome assessment, statistical analysis, interpretation of results, or publication decisions, and will not have access to unblinded individual-level data.

#### Sample size

As no previous studies have reported the effect of high-dose inhaled NO on CPIS outcomes in patients with severe pneumonia caused by MDR bacteria, the sample size was estimated using preliminary retrospective data from our center. These data included four patients who received high-dose inhaled NO and 11 patients who received standard treatment. The between-group difference in CPIS was 3 points, with a standard deviation of 4 points. Assuming a two-sided α of 0.05 and a β of 0.20 (80% power), at least 28 participants per group are required, for a total of 56 participants. Allowing a 10% dropout rate, the target sample size is 64 participants with confirmed MDR bacterial infection (32 per group). Assuming that 65% of patients with suspected MDR infection will have confirmed MDR bacterial infection, approximately 98 patients with suspected MDR infection will need to be enrolled.

#### Outcomes

##### Primary outcome

The primary outcome is the change in CPIS from baseline to day 7.

##### Secondary outcomes

 

1. In-hospital mortality, ICU mortality, and 28-day mortality.
2. Ventilator-free days, vasopressor-free days, antibiotic-free days, ICU-free days, and hospital-free days through day 28.
3. CPIS and Sequential Organ Failure Assessment (SOFA) scores on days 3 and 7.
4. Microbiological eradication rate on day 7.
5. Concentrations of citrullinated histone H3 and cell-free DNA.
6. Daily ratio of arterial oxygen partial pressure to fractional inspired oxygen (PaO_2_/FiO_2_) from days 1 to 7.

##### Safety outcomes

1. Incidence of MetHb levels ≥5%, ≥7%, and ≥10%.
2. Incidence of inhaled nitrogen dioxide (NO_2_) concentrations ≥3 ppm.
3. Serum creatinine levels from days 1 to 7.
4. Airway hyperreactivity events.
5. Incidence of acute kidney injury (AKI), including stages 1, 2, and 3, during hospitalization.

##### Analysis of populations

Three analysis populations will be defined: the modified intention-to-treat population with confirmed MDR pathogens (mITT-MDR), the per-protocol population with confirmed MDR pathogens (PP-MDR), and the safety population [24].

The safety population will include all randomized participants who receive at least one administration of study gas (NO or sham gas). Participants will be analyzed according to the treatment actually received, regardless of whether MDR bacterial infection is subsequently confirmed by antimicrobial susceptibility testing. Safety outcomes will be analyzed in the safety population.

The mITT-MDR population will include all participants who have at least one pathogen isolated from a baseline lower respiratory tract specimen that meets the prespecified MDR definition on antimicrobial susceptibility testing. Participants will be analyzed according to their randomized treatment assignment. The primary and secondary outcomes will be analyzed in the mITT-MDR population.

The PP-MDR population will include participants in the mITT-MDR population who complete at least 80% of the prespecified study-gas treatment sessions, have no major protocol deviations that could affect efficacy assessment, and complete assessment of the primary outcome. The PP-MDR analysis will be conducted only as a sensitivity analysis of the mITT-MDR analysis.

##### Data collection and management

Baseline data, underlying diseases, comorbidities, and the Acute Physiology and Chronic Health Evaluation II score on day 1 will be collected. Daily vital signs, Sequential Organ Failure Assessment score, and mechanical ventilation parameters will be monitored. Blood gas results will be collected before treatment, during the first 7 days, and at discharge. For patients receiving invasive mechanical ventilation at enrolment or on day 7, lower respiratory tract aspirates (endotracheal aspirates, ETAs) will be collected *via* the endotracheal tube using aseptic techniques. For patients who have been extubated by day 7 and can expectorate effectively, spontaneously expectorated deep sputum specimens will be collected. Blood samples collected on days 1 and 7 will be used to measure citrullinated histone H3 by enzyme-linked immunosorbent assay and cell-free DNA concentration.

Primary and secondary outcomes will be evaluated by independent assessors blinded to treatment allocation using standardized case report forms. To ensure data security, all source data will be anonymized using unique study IDs and stored on a password-protected server. Missing data will be minimized through standardized follow-up procedures. The amount, timing, and reasons for missing data will be summarized by randomized group.

##### Safety monitoring

During inhaled NO administration, NO2 will be continuously monitored and maintained below 3 ppm. MetHb levels will be measured 15 min after the initiation of each inhaled NO treatment session and at the end of each session. Renal function will be assessed daily using blood testing. An independent Data and Safety Monitoring Board (DSMB), comprising two external clinicians and one biostatistician, will review safety data every 6 months. No formal interim analysis of efficacy or futility and no sample-size re-estimation are planned. Serious adverse events will be reported within 24 h. The final database will be locked upon completion of the 30-day follow-up and resolution of all queries. Participant recruitment and study-gas treatment will be temporarily suspended, and an ad hoc safety review by the Data and Safety Monitoring Board (DSMB) will be initiated, if any of the following occurs:

1. Any unexpected death assessed as at least possibly related to study-gas treatment.
2. Two or more unexpected serious adverse events assessed as at least possibly related to study-gas treatment.
3. In the NO group, permanent discontinuation of study-gas treatment due to methemoglobin (MetHb) ≥7% or persistent inhaled NO_2_ concentrations >3 ppm in ≥10% of participants.
4. A clinically important and unexpected imbalance between groups in the incidence of serious adverse events, severe hypoxemia, or hemodynamic instability.
5. A major breach of blinding, major protocol deviation, or data-integrity issue that may jeopardize participant safety or seriously compromise trial validity.

##### Statistical analysis

Statistical analyses will be performed using R in RStudio. Changes in CPIS, 28-day ventilator-free days, vasopressor-free days, antibiotic-free days, ICU-free days, hospital-free days, CPIS and SOFA scores, PaO_2_/FiO_2_ ratios, and serum creatinine levels will be assessed for normality. Normally distributed continuous data will be expressed as mean ± SD and compared between groups using the independent-samples t test. Non-normally distributed continuous data will be expressed as median and interquartile range, M (Q1, Q3), and compared using the Wilcoxon rank-sum test. In-hospital mortality, ICU mortality, 28-day mortality, the incidences of MetHb levels ≥5%, ≥7%, and ≥10%, inhaled NO_2_ concentrations ≥3 ppm, airway hyperreactivity events, and AKI stages 1, 2, and 3 during hospitalization will be expressed as *n* (%) and compared between groups using the chi-square test or Fisher’s exact test, as appropriate. A P value less than 0.05 will be considered statistically significant.

##### Subgroup and sensitivity analyses

Prespecified subgroup analyses of the primary outcome will be conducted according to the presence or absence of septic shock, pneumonia type (community-acquired pneumonia [CAP] versus hospital-acquired pneumonia [HAP]), and concomitant use of inhaled antibiotics.

A sensitivity analysis of the primary outcome will be performed in the PP-MDR.

## Discussion

High-dose NO has anti-infective and oxygenation-improving effects. Several studies have suggested that inhaled NO can reduce viral load in patients with coronavirus disease 2019 and prevent postoperative pulmonary bacterial infection [3,8,10,11]. However, the effect of high-dose NO in Severe pneumonia infection has rarely been reported. This study focuses specifically on patients with severe pneumonia associated with MDR bacteria and will evaluate whether high-dose inhaled NO improves bacterial clearance, clinical outcomes, and mortality without increasing adverse reactions.

Mechanistically, high-dose NO may exert antibacterial activity by damaging bacterial DNA, proteins, and lipid structures [3], inhibiting biofilm formation by Pseudomonas aeruginosa and Klebsiella pneumoniae [16], and enhancing immune-cell phagocytosis of resistant bacteria [25]. A pathway involving neutrophil chemotaxis, activation, neutrophil extracellular trap release, and bacterial killing has also been proposed as a potential mechanism through which high-dose NO improves outcomes in severe pneumonia [16].

High-dose inhaled NO has been recommended for severe pneumonia in a Chinese expert consensus, primarily based on evidence from severe viral pneumonia [26]. Evidence for severe bacterial pneumonia remains limited. This study selects patients with MDR infection because these patients lack safe and effective treatment options, and conventional antibiotic therapy is often less effective or requires costly advanced antibiotics. If high-dose inhaled NO improves outcomes in patients with severe pneumonia associated with MDR bacteria, it may have important implications for antimicrobial resistance and patient prognosis.

This study also includes exploratory mechanistic assessments. Neutrophil number and percentage, citrullinated histone H3, and cell-free DNA concentration in plasma can reflect neutrophil extracellular trap activity and may provide preliminary clinical evidence for the proposed mechanism of action [27,28].

This study has several limitations. First, it is single-blind rather than double-blind. Because safety monitoring and clinical implementation make staff blinding difficult, medical staff will not be blinded, which may introduce bias. Second, this is a single-center study with a relatively small sample size, which may lead to selection bias and limit generalizability. Although the sample size calculation is based on the primary outcome, the study may be underpowered for mortality and other secondary outcomes.

In summary, this protocol provides a rigorous framework for evaluating high-dose inhaled NO in patients with severe pneumonia associated with MDR bacteria. By following protocol reporting standards, the study is expected to provide operational evidence to guide the optimization of treatment practice for patients with severe pneumonia associated with MDR bacteria.

## Supplementary Material

Table S1.docx

## Data Availability

The full protocol, dataset, statistical analysis plan, and informed consent materials will be available from the corresponding author after formal publication of this trial. No new data was generated or analyzed for the present protocol.
